# Two-Dimensional Spectroscopy of Open Quantum Systems: Nonequilibrium Green’s Function Formulation

**DOI:** 10.1021/acs.jpclett.4c03597

**Published:** 2025-02-18

**Authors:** Haoran Sun, Upendra Harbola, Shaul Mukamel, Michael Galperin

**Affiliations:** † Department of Chemistry & Biochemistry, University of California San Diego, La Jolla, California 92093, United States; ‡ Department of Inorganic and Physical Chemistry, Indian Institute of Science, Bangalore 560012, India; ¶ Department of Chemistry, University of California Irvine, Irvine, California 92697, United States; § School of Chemistry, Tel Aviv University, Tel Aviv 6997801, Israel

## Abstract

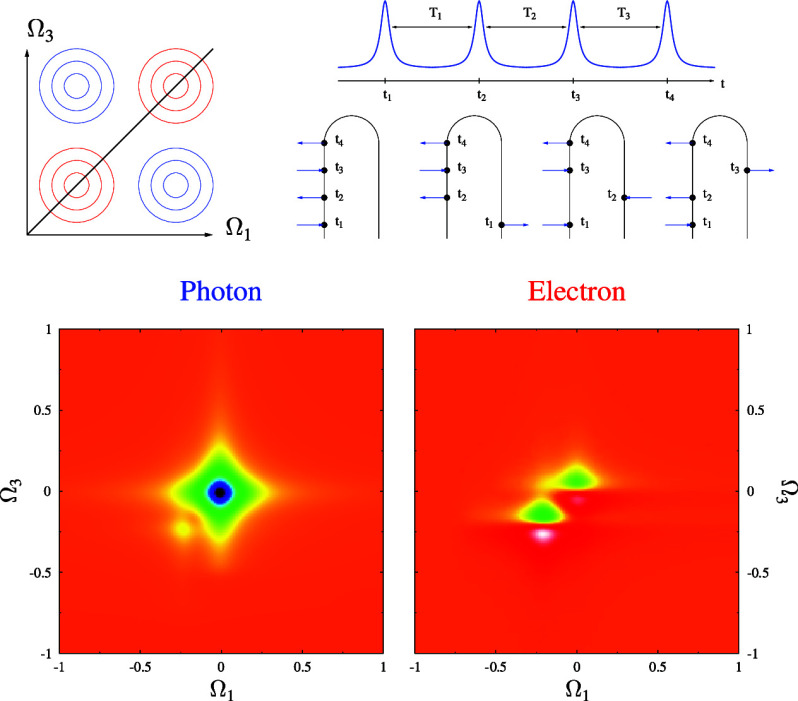

Two-dimensional spectroscopy is examined for open quantum systems featuring multiple simultaneously measurable fluxes. Specifically, we explore a junction where optical measurements of photon flux are paired with concurrent transport measurements of electron currents. The theory of two-dimensional spectroscopy for both fluxes is developed using a nonself-consistent nonequilibrium Green’s function formulation. Theoretical derivations are demonstrated through numerical simulations within a generic junction model.

Interaction of light with matter is one of the central research areas in chemistry. Traditionally, spectroscopy serves as a tool to investigate a system’s responses to external perturbations by radiation fields. Initially, studies concentrated on molecules in the gas phase. However, with advancements in experimental techniques, optical spectroscopy investigations have expanded to include open molecular systems (molecules at surfaces, molecules at interfaces, molecules in cavities).^1−3^ Contrary to gas-phase experiments, photon flux is not the only measurable response in such systems. Electron transfer and transport represent another quantifiable characteristic in open molecular systems. Similar to optical spectroscopy, which focuses on photon flux, current spectroscopy employs bias-induced electron flux to investigate molecular properties. For instance, this includes resonant^4,5^ and off-resonant^6−10^ inelastic electron tunneling spectroscopy. Research that concentrates on the interplay between light (photon) and matter (electron) responses in single molecule junctions is termed optoelectronics.^11,12^

Molecular optoelectronics encompasses several research directions originally focused on one type of response due to the other type of perturbation. For instance, this includes bias-induced light^13−15^ (including light emission above threshold when emitting photon energy is higher that energy available due to bias^16−19^) and light-induced current (photocurrent)^20−25^ (including control of the current by light^26−28^). The further development of laser techniques at the nanoscale has enabled the simultaneous measurement of photon and electron fluxes in single-molecule junctions.^29−34^

Multidimensional optical spectroscopy serves as a tool for investigating the electronic structure and dynamics of complex molecular systems in the condensed phase. Measurements that extend this technique to electron flux, known as two-dimensional (2D) photocurrent spectroscopy, were recently proposed in the literature^35,36^ and applied to decompose the photovoltaic response into single exciton and biexciton contributions,^37^ revealing ultrafast hole transfer in solar cells,^38^ probing carrier diffusion and two-body recombination processes in layered perovskites,^39^ and identifying incoherent mixing effects in semiconductors.^40^ Reviews of the technique can be found in refs (41−44).

Multidimensional spectroscopy that simultaneously measures electron and photon fluxes represents the natural next step. The standard experimental setup employs a sequence of four laser pulses while measuring the system’s response to perturbation as a function of time delays *T*_1_, *T*_2_, and *T*_3_ between pulses. The response is recorded as a 2D diagram of integrated flux vs Ω_1_ and Ω_3_ (Fourier transforms of sequences of measurements in delays *T*_1_ and *T*_3_), providing information on the quantum coherences within the system. A sequence of diagrams for different *T*_2_ offers insights into the dynamics of these coherences (see Figure 1).

**Figure 1:**
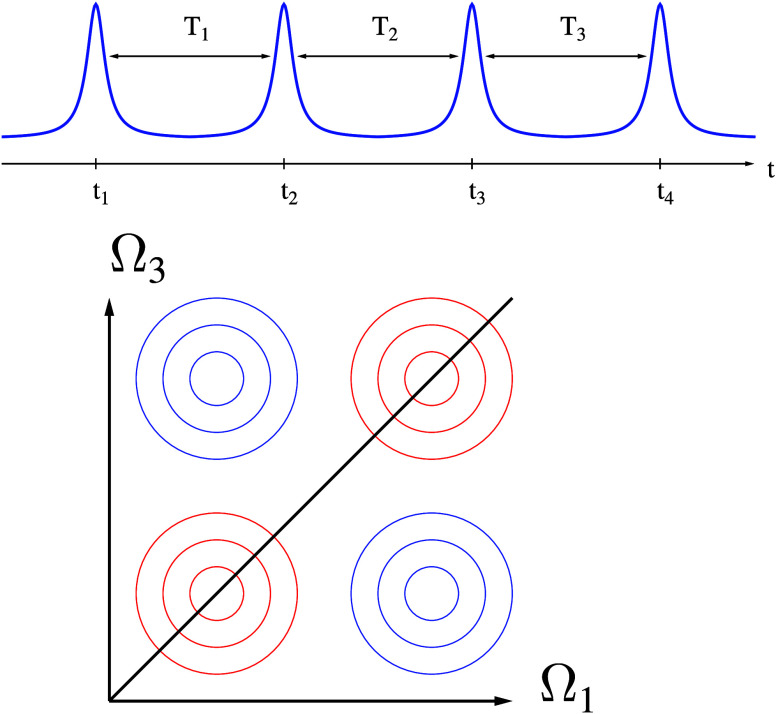
Sketch of two-dimensional spectroscopy signal.

Here, we present a theoretical description of 2D optical spectroscopy in open quantum systems, where both photon and electron fluxes are measured simultaneously. Our recent formulation of nonlinear spectroscopy in open quantum systems^45^ serves as the starting point. The quantum description of the radiation field is simplified to a classical treatment of laser pulses, establishing a connection to the Feynman diagrams of the quantum treatment. We derive explicit expressions for the multidimensional signals resulting from photon and electron fluxes, following the formulation of ref (45), when the signal is modeled as a fourth-order response function. Finally, we illustrate the limitations of the approach and discuss potential alternatives.

We note that, in contrast to previous NEGF-based multidimensional considerations,^46^ where photon flux was derived from the bare perturbation expansion of nonlinear optical spectroscopy, we now employ a newly developed NEGF-based theory that allows for the evaluation of both photon and electron fluxes in a consistent manner. Additionally, we observe that the description of multidimensional cavity spectroscopy, taking into account the strong light-matter interaction, necessitates modifications to the latter theory and is reserved for future research.

This Letter is structured as follows. After introducing a model of an open quantum system and discussing the treatment of its responses within the nonequilibrium Green’s function (NEGF) method, we derive expressions for 2D signals obtained through nonlinear spectroscopy methods for photon and electron fluxes. Simple generic models are utilized to present numerical results that illustrate our findings. Our presentation concludes with a discussion of results and future research directions.

We consider molecular system *M*, coupled to two contacts (*L* and *R*), each at its own equilibrium, and driven by external classical field *E*(*t*). The Hamiltonian of the model is
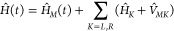
1where *Ĥ*_*M*_(*t*) and *Ĥ*_*K*_ describe, respectively, driven molecular system and contact *K*. *V̂*_*MK*_ couples between the subsystems. Explicit expressions for the Hamiltonians are
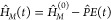
2
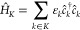
3
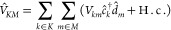
4Here, *d̂*_*m*_^†^ (*d̂*_*m*_) and *ĉ*_*k*_^†^ (*ĉ*_*k*_) creates (annihilates) electron in molecular orbital *m* and state *k* of contacts, respectively.
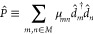
5is the molecular dipole operator, and *Ĥ*_*M*_^(0)^ describes the molecular system in the absence of driving.

We analyze electron *I*_*e*_^*K*^(*t*) (*K* = *L*, *R*) and photon *I*_*p*_(*t*) fluxes. Their explicit expressions are^45,47^
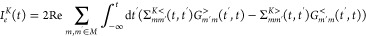
6
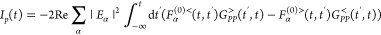
7Here, *G* and *G*_*PP*_ are the single and two-particle Green’s functions^45^
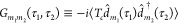
8
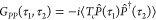
9and Σ^*K*^ is the electron self-energy due to coupling to contact *K*
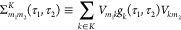
10where
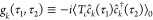
11is the Green’s function of free electron in state *k* of the contacts (subscript “0” indicates free evolution in the contacts).

Note that, similar to standard nonlinear optical spectroscopy, derivation of photon flux is based on quantum description of radiation field. Correspondingly, *F*_α_^(0)^ in eq 7 is Green’s function of free photons in mode α of the field,
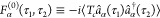
12and *â*_α_^†^ (*â*_α_) creates (destroys) photons in the mode α.

In our model (eq 1), the radiation field is treated classically (as external driving force). Correspondence between quantum and classical descriptions can be obtained by the following substitution:
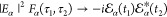
13where *t*_1,2_ are physical times corresponding to contour variables τ_1,2_, α is any mode of the radiation field, and
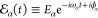
14is the value of the classical field mode α at time *t*. We note that not all Feynman diagrams related to quantum considerations contribute to the treatment of classical radiation fields. For instance, diagrams corresponding to virtual quantum processes should be disregarded. Indeed, classical fields do not accommodate virtual processes. This is evident in the fact that, for example, the fourth-order diagram in Figure 6c of ref (45) separates into two independent second-order processes when transitioning to a classical description.

2D spectroscopy relies on the system’s response to a sequence of four monochromatic pulses at an ordered set of times *t*_1_ < *t*_2_ < *t*_3_ < *t*_4_ (see Figure 1). Traditional spectroscopy considers photon flux as a fourth-order process in light-matter interaction (third-order response function).^48^

The analogue of such treatment in open systems begins by formulating fourth-order expressions for electron and photon fluxes (see eqs 6 and 7) within the NEGF bare diagrammatic expansion, as introduced in ref (45). These expressions are derived by substituting the fourth-order contribution to *G* into eq 6 and second-order contribution of *G*_*PP*_ into eq 7 (see eqs (12), (28), (A1), (A2), and (B1) of ref (45)). Explicit expressions for the fluxes, when the radiation field is treated classically, are provided in the Supporting Information.

System response in 2D spectroscopy is characterized by the Fourier transform in delay times *T*_1_ and *T*_3_ (for fixed *T*_2_) of the integrated flux,
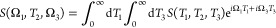
15Here, *S*(*T*_1_, *T*_2_, *T*_3_) is the total charge passed at interface *K* or the total number of photons,
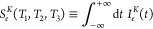
16
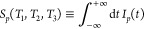
17Evaluation of explicit expressions relies on exact expressions for electron and photon fluxes and various observations and assumptions (see the Supporting Information for details). This leads to the following expressions for the 2D signals:
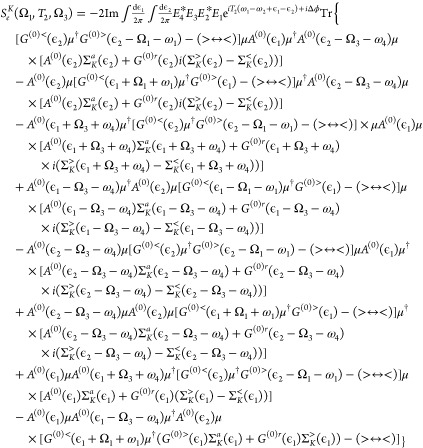
18for 2D electron spectroscopy, and
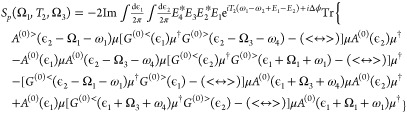
19for 2D photon spectroscopy. Here, the symbols >, <, *r*, and *a* represent the greater, lesser, retarded, and advanced projections, respectively.^49^ Δϕ ≡ ϕ_1_ – ϕ_2_ + ϕ_3_ – ϕ_4_, and *A*_*mm*′_^(0)^(ϵ) is the spectral function:
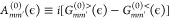
20 In eqs 18 and 19, restriction is fulfilled:
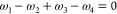
21Equations 18 and 19 are key results of our analysis.

We now illustrate the 2D spectroscopy signals described by eqs 18 and 19 for a generic junction model. We consider a molecule with two energetically distinct optical transitions between the ground state and two excited states. The ground state of the molecule is strongly coupled to both contacts. One of excited states is coupled to contact on the left and the other on the right. Electron transfer is possible between the two excited states (see Figure 2):
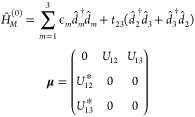
22For simplicity, we assume a wide-band approximation for self-energies resulting from coupling to contacts.

**Figure 2:**
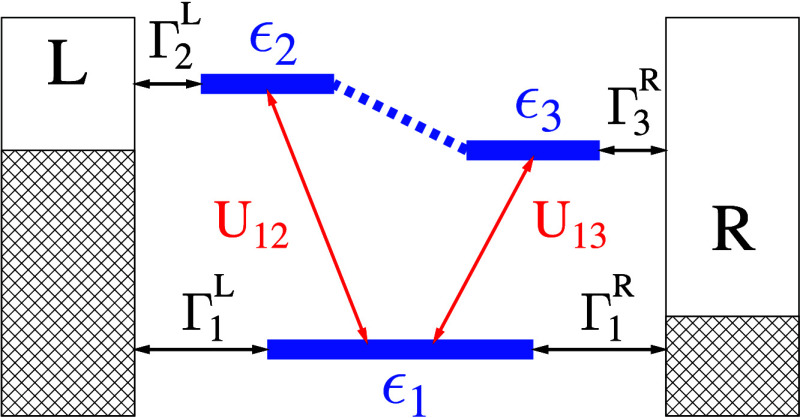
Generic junction model.

Parameters of the simulations are as follows. The temperature is *T* = 0.025, and the positions of levels are ϵ_1_ = −0.5, ϵ_2_ = 0.5, ϵ_3_ = 0.3. The electron transfer matrix element is *t* = 0.1. Escape rates to contacts are Γ_1_^*L*^ = Γ_1_^*R*^ = 0.1 and Γ_2_^*L*^ = Γ_3_^*R*^ = 0.01. The light-matter coupling strength is *U*_12_*E*_α_ = *U*_13_*E*_α_ = 0.01. Simulations are conducted on an energy grid spanning range from −3 to 3 with a step of 0.001. The Fermi energy (*E*_F_) is taken as the origin (*E*_*F*_ = 0), and a bias *V* is applied symmetrically, μ_*L*,*R*_ = *E*_F_ ± | *e*| *V*/2. Here, energy is expressed in terms of an arbitrary unit of energy *E*_0_. The resulting signal (photon and electron fluxes) is presented in terms of the flux unit *I*_0_ = 1/*t*_0_, where *t*_0_ = 1/*E*_0_ is the unit of time.

Figure 3 presents the results of a simulation using the model depicted in Figure 2 at equilibrium for a range of delay times *T*_2_. Initially, *T*_2_ = 0, the optical response *S*_*p*_ yields the typical 2D signal characterized by two distinct transitions (peaks on diagonal) and coherences between them (off-diagonal peaks). Current spectroscopy does not have any information because, at *T*_2_ = 0, the optical excitation has not yet influenced the current (*I*_*L*_ = *I*_*R*_ = 0). As the time delay increases, two processes occur: dephasing due to coupling with the contacts and Rabi oscillation affecting populations of the excited states ϵ_2_ and ϵ_3_. Additionally, the populations of the excited states generate electron current into the left and right contacts. Therefore, in the optical signal, the coherences between the two excitations diminish, and the relative intensity of the two peaks is determined by the Pauli blockade of one of the transitions due to the Rabi oscillation of the excited states’ populations. A 2D current signal emerges at finite *T*_2_ with *S*_*L*_^*e*^ and *S*_*R*_^*e*^ primarily providing local information (that is, information about the transition nearest to the corresponding contact). It is important to note that increasing *T*_2_ reduces overall signal strength (not shown) as the system approaches a steady state following the disturbance caused by the first pair of pulses.

**Figure 3:**
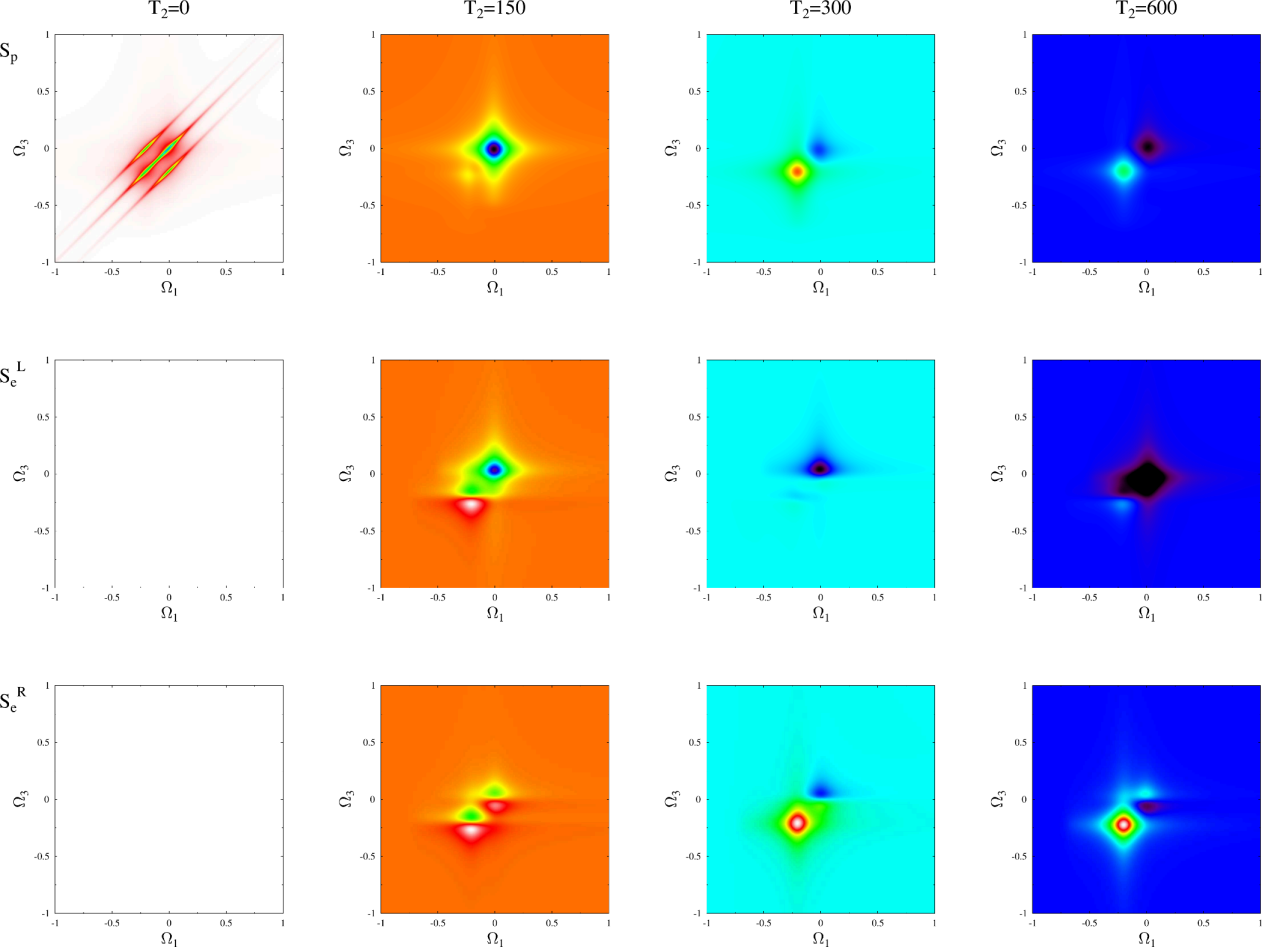
2D spectroscopy for the mode of Figure 2 at zero bias. Shown are *S*_*p*_ (top row), *S*_*L*_^*e*^ (middle row), and *S*_*R*_^*e*^ (bottom row) for *T*_2_ = 0 (first column), 150 (second column), 300 (third column), and 600 (last column).

Figure 4 presents the results of a simulation using the model depicted in Figure 2 for biased junction, where | *e*| *V* = 2, across a range of delay times *T*_2_. The qualitative behavior mirrors that of the equilibrium case, with bias-induced depletion of the ground state and the population of excited states influencing the strength of the signal.

**Figure 4:**
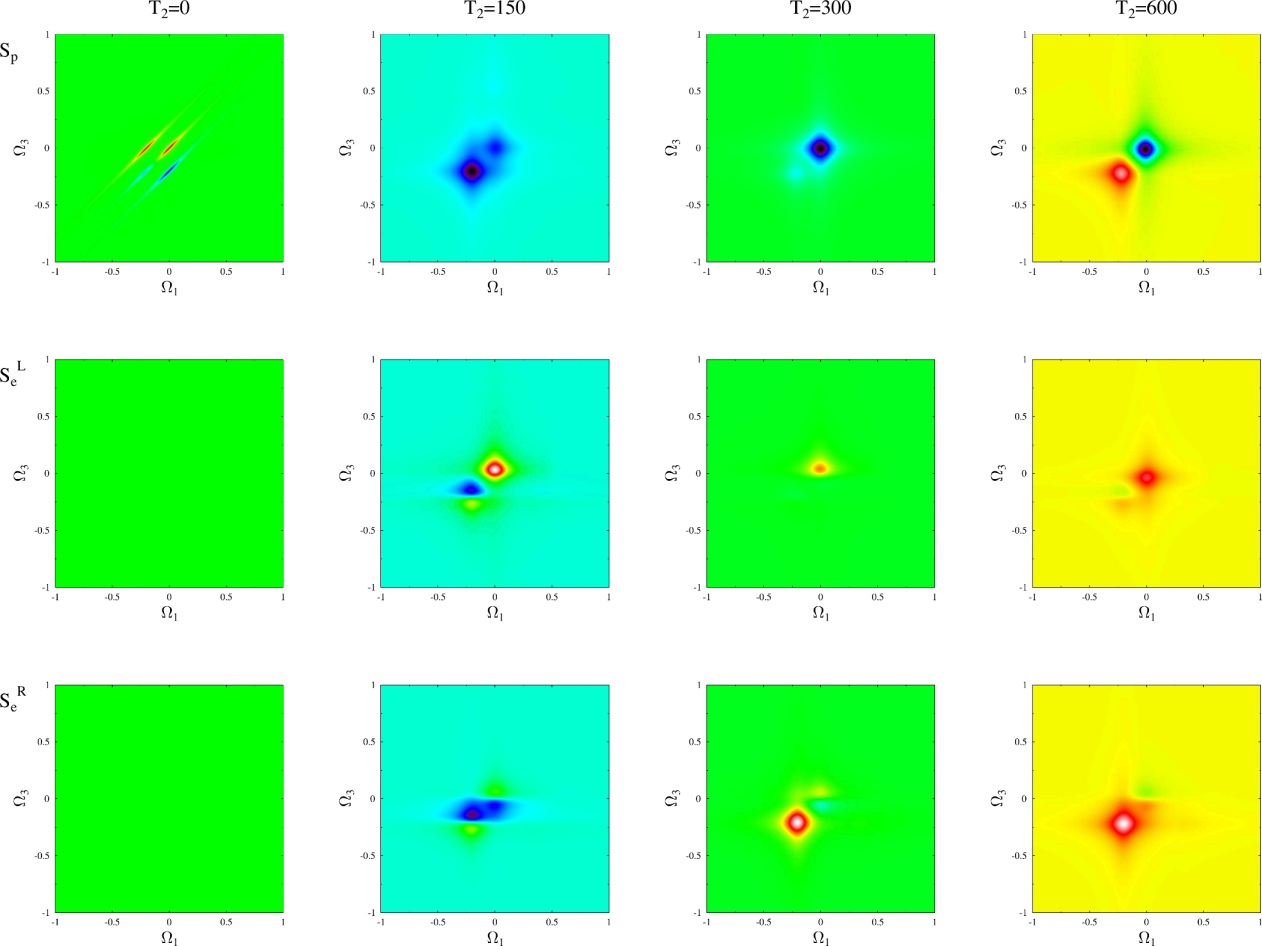
2D spectroscopy for the mode of Figure 2 at bias |*e*|*V* = 20. Shown are *S*_*p*_ (top row), *S*_*e*_^*L*^ (middle row), and *S*_*e*_^*R*^ (bottom row) for *T*_2_ = 0 (first column), 150 (second column), 300 (third column), and 600 (last column).

Thus, the photon flux and electron current measured within the multidimensional spectroscopy setup provide complementary information, with photon flux offering insights into the system as a whole, while electron current delivers a more localized description (it primarily reveals the behavior of degrees of freedom near the contact). This creates the possibility of using, for example, an STM tip as a local probe for correlations within the system in the multidimensional spectroscopy setup.

Finally, we note that, for realistic parameters, the traditional perturbation theory treatment of 2D spectroscopy may sometimes be inaccurate, because it overlooks the effect of the radiation field on the electronic structure of the system. Figure 5 illustrates this point by comparing the results of a full NEGF simulation (solid line, blue) to a fourth-order treatment of light-matter interaction. The coupling to the field is μ*E*_α_ = 0.5, while other parameters remain consistent with previous simulations. It is evident that the full calculation yields results that deviate significantly from the fourth-order perturbation theory treatment. In such cases, the generalization of the traditional treatment for multidimensional optical spectroscopy presented above is insufficient, and a full flux simulation is required.

**Figure 5:**
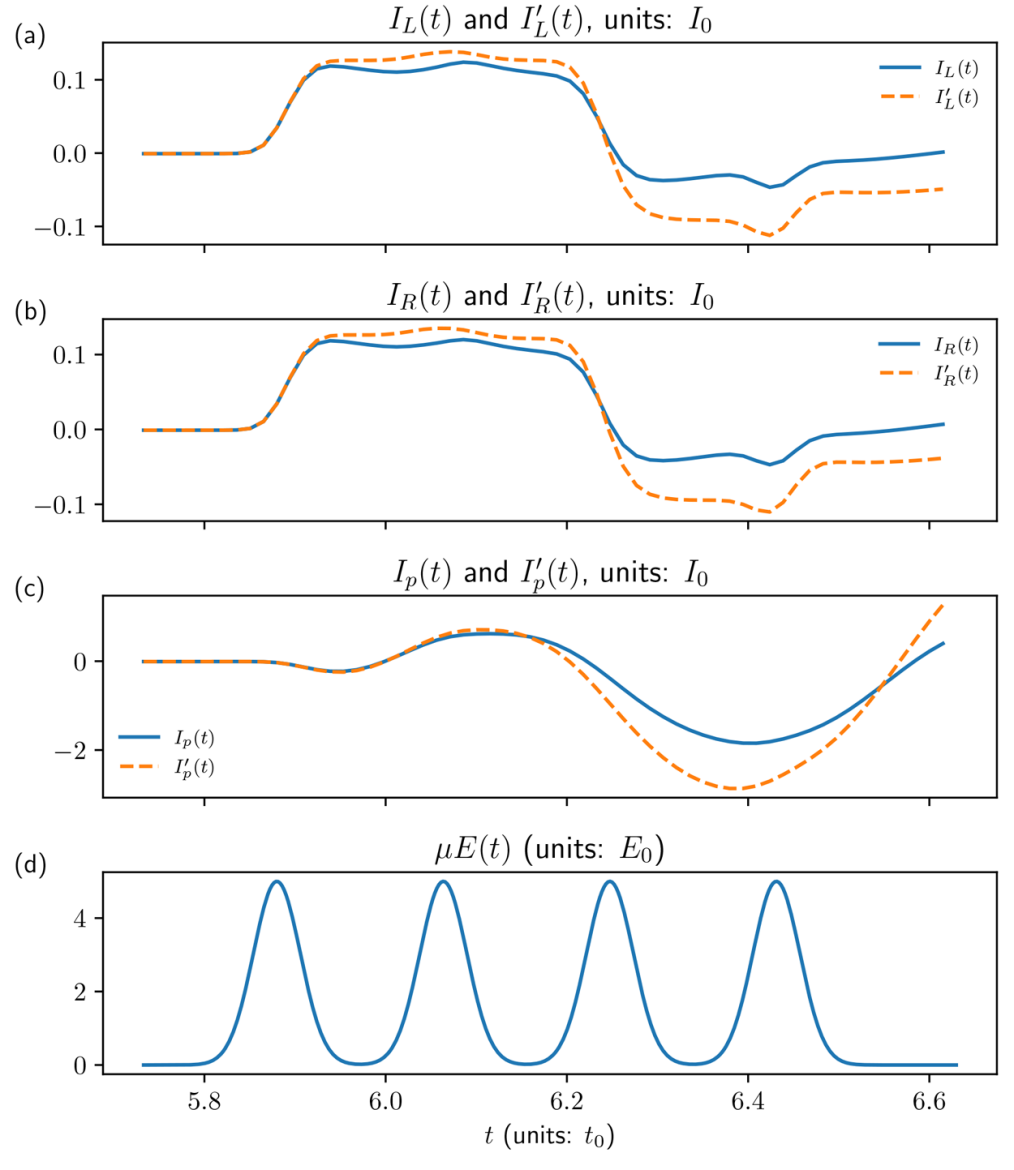
Electron and photon fluxes induced by sequence of laser pulses for junction model of Figure 2 at zero bias. Shown are results of full (solid line, blue) and fourth order in light-mater interaction (dashed line, red) NEGF simulations for (a) electron current at interface *L*, (b) electron current at interface *R*, and (c) photon flux. Panel (d) shows the sequence of driving laser pulses.

We now present a summary of our findings. The development of experimental techniques at the nanoscale has made it possible to conduct simultaneous measurements of optical (photon flux) and transport (electron current) characteristics of open nonequilibrium quantum systems. The theoretical description of such experiments necessitates the generalization of standard tools in nonlinear optical spectroscopy. In particular, formulations of experimentally measurable bias-induced electroluminescence and Raman spectroscopy in single-molecule junctions have been discussed in the literature.^15,50−52^

2D spectroscopy is a technique that allows for the study of system responses and coherences among its degrees of freedom in complex molecular systems in the condensed phase. Optical spectroscopy utilizes photon flux to analyze system responses.^46,53^ Recently, experimental measurements of electron current as the signal for the 2D spectroscopy analysis were reported in the literature.^35,36^ The natural next step is to conduct 2D spectroscopy simultaneously in both photon and electron fluxes.

Because photon flux and electron current are interdependent in an open quantum system, the theoretical consideration of simultaneous 2D spectroscopy necessitates treating both fluxes on equal footing. Here, we present a theoretical formulation of 2D spectroscopy in open quantum systems with simultaneous measurement of photon flux and electron current. This formulation is based on the recently introduced non-self-consistent nonequilibrium Green’s function approach to nonlinear spectroscopy.^45^ Starting from this quantum mechanical framework, we consider the transition to a classical description of the radiation field and apply the methodology to derive the 2D spectroscopy signals. The approach consistently accounts for all the fluxes in the system, which, in particular, preserves conservation laws. Expressions for the 2D signals are derived as fourth-order contributions to the fluxes from light–matter interactions.

We illustrate the theoretical formulation with numerical simulations for a generic model of a molecular junction. We find that the traditional optical signal of 2D spectroscopy, *S*_*p*_, along with the electron fluxes, *S*_*e*_^*L*^ and *S*_*e*_^*R*^, provides complementary information about the system when detected simultaneously. In particular, for the model in Figure 2, *S*_*p*_ yields overall information on optical transitions, while *S*_*e*_^*L*^ and *S*_*e*_^*R*^ represent the corresponding local contributions. Additionally, when excited by a laser field, the optical signal is available at earlier times than that caused by the electron flux. Finally, we note that, for realistic parameters, the traditional perturbation theory treatment of 2D spectroscopy may sometimes be inaccurate, because it overlooks the effect of the radiation field on the electronic structure of the system. In such cases, full flux simulation is required.

## Supporting Information Available

Fourth-order expressions for electron and photon fluxes and details of derivation of the two-dimensional signals (PDF)
