# Supplementary material for: Two-Dimensional Spectroscopy of Open Quantum Systems: Nonequilibrium Green’s Function Formulation
Source: J Phys Chem Lett. 2025 Feb 18;16(8):2008–15. doi: 10.1021/acs.jpclett.4c03597 (PMC11873975; doi:10.1021/acs.jpclett.4c03597)
Supplement: Supplementary file 1 — jz4c03597_si_001.pdf [file jz4c03597_si_001.pdf]

Supporting Information: Two-dimensional spectroscopy of open quantum systems: Nonequilibrium Green's function formulation

Haoran Sun,† Upendra Harbola,‡ Shaul Mukamel,¶ and Michael Galperin*,§

†Department of Chemistry & Biochemistry, University of California San Diego, La Jolla, CA 92093, USA

‡Department of Inorganic and Physical Chemistry, Indian Institute of Science, Bangalore 560012, India

¶Department of Chemistry, University of California Irvine, Irvine, CA 92697, USA

§School of Chemistry, Tel Aviv University, Tel Aviv 69978, Israel

E-mail: mgalperin@tauex.tau.ac.il

Fourth order expressions for fluxes

Fourth order expression for electron flux is

$$
\begin{aligned}
I_e^{K\,(4)}(t) = 2\,\mathrm{Re}\int_c d\tau' \int_c d\tau_a \int_c d\tau_b \int_c d\tau_c \int_c d\tau_d\, \mathcal{E}^*(t_d)\,\mathcal{E}(t_c)\,\mathcal{E}^*(t_b)\,\mathcal{E}(t_a) \\
\mathrm{Tr}\Big[G^{(0)}(t,\tau_a)\,\mu^\dagger\,G^{(0)}(\tau_a,\tau_c)\,\mu^\dagger\,G^{(0)}(\tau_c,\tau_b)\,\mu\;\;G^{(0)}(\tau_b,\tau_d)\,\mu\;\;G^{(0)}(\tau_d,\tau')\,\Sigma^K(\tau',t) \\
+\,G^{(0)}(t,\tau_a)\,\mu^\dagger\,G^{(0)}(\tau_a,\tau_d)\,\mu\;\;G^{(0)}(\tau_d,\tau_b)\,\mu\;\;G^{(0)}(\tau_b,\tau_c)\,\mu^\dagger\,G^{(0)}(\tau_c,\tau')\,\Sigma^K(\tau',t) \\
+\,G^{(0)}(t,\tau_b)\,\mu\;\;G^{(0)}(\tau_b,\tau_c)\,\mu^\dagger\,G^{(0)}(\tau_c,\tau_a)\,\mu^\dagger\,G^{(0)}(\tau_a,\tau_d)\,\mu\;\;G^{(0)}(\tau_d,\tau')\,\Sigma^K(\tau',t) \\
+\,G^{(0)}(t,\tau_b)\,\mu\;\;G^{(0)}(\tau_b,\tau_d)\,\mu\;\;G^{(0)}(\tau_d,\tau_a)\,\mu^\dagger\,G^{(0)}(\tau_a,\tau_c)\,\mu^\dagger\,G^{(0)}(\tau_c,\tau')\,\Sigma^K(\tau',t) \\
+\,G^{(0)}(t,\tau_a)\,\mu^\dagger\,G^{(0)}(\tau_a,\tau_b)\,\mu\;\;G^{(0)}(\tau_b,\tau_c)\,\mu^\dagger\,G^{(0)}(\tau_c,\tau_d)\,\mu\;\;G^{(0)}(\tau_d,\tau')\,\Sigma^K(\tau',t) \\
+\,G^{(0)}(t,\tau_a)\,\mu^\dagger\,G^{(0)}(\tau_a,\tau_b)\,\mu\;\;G^{(0)}(\tau_b,\tau_d)\,\mu\;\;G^{(0)}(\tau_d,\tau_c)\,\mu^\dagger\,G^{(0)}(\tau_c,\tau')\,\Sigma^K(\tau',t) \\
+\,G^{(0)}(t,\tau_b)\,\mu\;\;G^{(0)}(\tau_b,\tau_a)\,\mu^\dagger\,G^{(0)}(\tau_a,\tau_c)\,\mu^\dagger\,G^{(0)}(\tau_c,\tau_d)\,\mu\;\;G^{(0)}(\tau_d,\tau')\,\Sigma^K(\tau',t) \\
+\,G^{(0)}(t,\tau_b)\,\mu\;\;G^{(0)}(\tau_b,\tau_a)\,\mu^\dagger\,G^{(0)}(\tau_a,\tau_d)\,\mu\;\;G^{(0)}(\tau_d,\tau_c)\,\mu^\dagger\,G^{(0)}(\tau_c,\tau')\,\Sigma^K(\tau',t) \\
+\,G^{(0)}(t,\tau_a)\,\mu^\dagger\,G^{(0)}(\tau_a,\tau_c)\,\mu^\dagger\,G^{(0)}(\tau_c,\tau_d)\,\mu\;\;G^{(0)}(\tau_d,\tau_b)\,\mu\;\;G^{(0)}(\tau_b,\tau')\,\Sigma^K(\tau',t) \\
+\,G^{(0)}(t,\tau_a)\,\mu^\dagger\,G^{(0)}(\tau_a,\tau_d)\,\mu\;\;G^{(0)}(\tau_d,\tau_c)\,\mu^\dagger\,G^{(0)}(\tau_c,\tau_b)\,\mu\;\;G^{(0)}(\tau_b,\tau')\,\Sigma^K(\tau',t) \\
+\,G^{(0)}(t,\tau_b)\,\mu\;\;G^{(0)}(\tau_b,\tau_c)\,\mu^\dagger\,G^{(0)}(\tau_c,\tau_d)\,\mu\;\;G^{(0)}(\tau_d,\tau_a)\,\mu^\dagger\,G^{(0)}(\tau_a,\tau')\,\Sigma^K(\tau',t) \\
+\,G^{(0)}(t,\tau_b)\,\mu\;\;G^{(0)}(\tau_b,\tau_d)\,\mu\;\;G^{(0)}(\tau_d,\tau_c)\,\mu^\dagger\,G^{(0)}(\tau_c,\tau_a)\,\mu^\dagger\,G^{(0)}(\tau_a,\tau')\,\Sigma^K(\tau',t)\Big]
\end{aligned}
\tag{S1}
$$

Fourth order expression for photon flux is

$$I_p^{(4)}(t) = 2\,\mathrm{Re}\int_c d\tau_a \int_c d\tau_b \int_c d\tau_c\, \mathcal{E}^*(t)\,\mathcal{E}(t_c)\,\mathcal{E}^*(t_b)\,\mathcal{E}(t_a) \tag{S2}$$
$$\mathrm{Tr}\Big[G^{(0)}(t,\tau_a)\,\mu^\dagger\,G^{(0)}(\tau_a,\tau_b)\,\mu\;\;G^{(0)}(\tau_b,\tau_c)\,\mu^\dagger\,G^{(0)}(\tau_c,t)\,\mu$$
$$+\,G^{(0)}(t,\tau_b)\,\mu\;\;G^{(0)}(\tau_b,\tau_a)\,\mu^\dagger\,G^{(0)}(\tau_a,\tau_c)\,\mu^\dagger\,G^{(0)}(\tau_c,t)\,\mu$$
$$+\,G^{(0)}(t,\tau_c)\,\mu^\dagger\,G^{(0)}(\tau_c,\tau_a)\,\mu^\dagger\,G^{(0)}(\tau_a,\tau_b)\,\mu\;\;G^{(0)}(\tau_b,t)\,\mu$$
$$+\,G^{(0)}(t,\tau_c)\,\mu^\dagger\,G^{(0)}(\tau_c,\tau_b)\,\mu\;\;G^{(0)}(\tau_b,\tau_a)\,\mu^\dagger\,G^{(0)}(\tau_a,t)\,\mu$$
$$+\,G^{(0)}(t,\tau_b)\,\mu\;\;G^{(0)}(\tau_b,\tau_c)\,\mu^\dagger\,G^{(0)}(\tau_c,\tau_a)\,\mu^\dagger\,G^{(0)}(\tau_a,t)\,\mu$$
$$+\,G^{(0)}(t,\tau_a)\,\mu^\dagger\,G^{(0)}(\tau_a,\tau_c)\,\mu^\dagger\,G^{(0)}(\tau_c,\tau_b)\,\mu\;\;G^{(0)}(\tau_b,t)\,\mu\Big]$$

Evaluation of multi-dimensional signals S_e^K and S_p starts from expressions for the fluxes, Eqs. (S1) and (S2), and employs the following approximations.

1. One must establish a connection between t_a, t_b, t_c, t_d (the physical times corresponding to contour variables $\tau_a, \tau_b, \tau_c, \tau_d$) in the expressions for the fluxes, Eqs. (S1) and (S2), and t_1, t_2, t_3, t_4 of the pulses. For the photon flux $I_p(t)$, it is natural to identify t_4 with the latest time of the signal t, then t_2 corresponds to t_b while the pair t_1, t_3 can be identified with t_a and t_c in any ordering. For the electron flux $I_e^K(t)$, the pair t_4, t_2 is identified with t_b and t_d in any order, while the pair t_3, t_1 corresponds to t_c and t_a, also in any ordering.

2. Contour integrations in Eqs. (S1) an (S2) imply all possible placements of variables $\tau_{a,b,c,d}$ on the Keldysh contour. However, condition $t_4 > t_3 > t_2 > t_1$ restricts placement possibilities to projections (double-sided Feynman diagrams) presented in Fig. S1. Note that for photon flux only the projections in Fig. S1a (or only in Fig. S1b) need to be taken into account. This follows from the choice $t = t_4$ and cyclic property of trace. Additionally, for electron flux, there is also integration in τ'. Thus, for each line in expression (S1) one must consider $3 \times 2^4 = 48$ projections, while each line in Eq.(S2)

yields $2^3 = 8$ projections.

3. To simplify the expressions, spectroscopic analysis assumes that laser pulses are local in time (with a pulse width of zero). This eliminates the corresponding integrals in the final results.

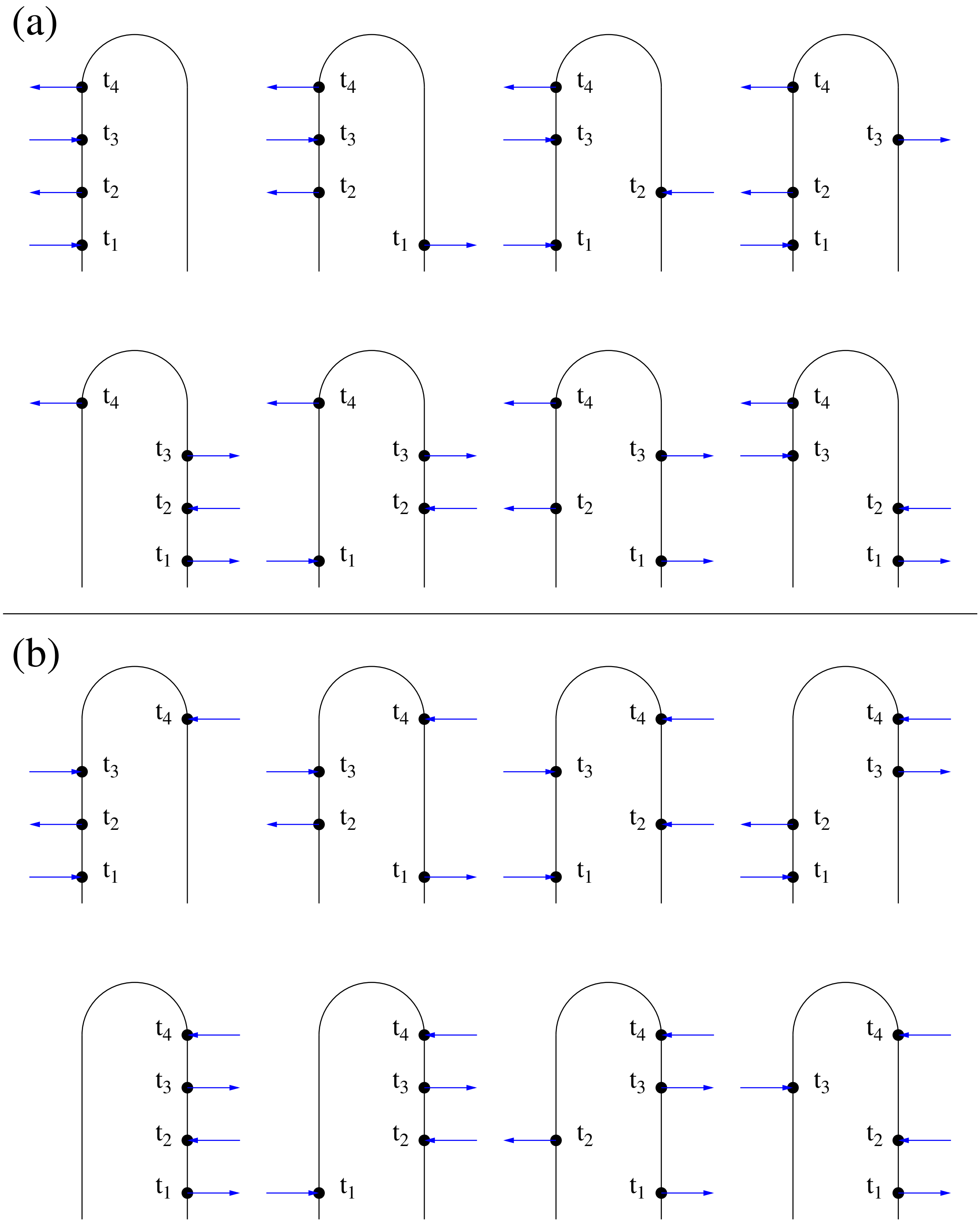

Figure S1: Projections (double-sided Feynman diagrams) contributing to the two-dimensional signal. S_p signal is given by contributions of panel (a). S_e^K ($K = L, R$) signal requires accounting for contributions of panels (a) and (b).
